# Computational modelling of energy balance in individuals with Metabolic Syndrome

**DOI:** 10.1186/s12918-019-0705-z

**Published:** 2019-02-26

**Authors:** Yvonne J. W. Rozendaal, Yanan Wang, Peter A. J. Hilbers, Natal A. W. van Riel

**Affiliations:** 1 Department of Biomedical Engineering, Eindhoven University of Technology, Eindhoven, The Netherlands; 2 Department of Pediatrics, Section Molecular Genetics, University Medical Center Groningen, University of Groningen, Groningen, The Netherlands; 3 Department of Medicine, Division of Endocrinology, Leiden University Medical Center, Leiden, The Netherlands; 4 Department of Vascular Medicine, Amsterdam University Medical Centers, University of Amsterdam, Amsterdam, The Netherlands

**Keywords:** Metabolic syndrome, Energy expenditure, Obesity, Patient-specific, Computational modelling, Heterogeneity, Lipid metabolism, Cold exposure, Brown adipose tissue

## Abstract

**Background:**

A positive energy balance is considered to be the primary cause of the development of obesity-related diseases. Treatment often consists of a combination of reducing energy intake and increasing energy expenditure. Here we use an existing computational modelling framework describing the long-term development of Metabolic Syndrome (MetS) in APOE3L.CETP mice fed a high-fat diet containing cholesterol with a human-like metabolic system. This model was used to analyze energy expenditure and energy balance in a large set of individual model realizations.

**Results:**

We developed and applied a strategy to select specific individual models for a detailed analysis of heterogeneity in energy metabolism. Models were stratified based on energy expenditure. A substantial surplus of energy was found to be present during MetS development, which explains the weight gain during MetS development. In the majority of the models, energy was mainly expended in the peripheral tissues, but also distinctly different subgroups were identified.

In silico perturbation of the system to induce increased peripheral energy expenditure implied changes in lipid metabolism, but not in carbohydrate metabolism. In silico analysis provided predictions for which individual models increase of peripheral energy expenditure would be an effective treatment.

**Conclusion:**

The computational analysis confirmed that the energy imbalance plays an important role in the development of obesity. Furthermore, the model is capable to predict whether an increase in peripheral energy expenditure – for instance by cold exposure to activate brown adipose tissue (BAT) – could resolve MetS symptoms.

**Electronic supplementary material:**

The online version of this article (10.1186/s12918-019-0705-z) contains supplementary material, which is available to authorized users.

## Background

A positive energy balance is a major contributor to the development of obesity and its related disorders such as the Metabolic Syndrome (MetS) [1–4]. The Metabolic Syndrome is characterized by the joint manifestation of obesity with hyperglycemia, insulin resistance, dyslipidemia and/or hypertension [5–8]. MetS imposes severe health risks and complications and increases the risk to develop other diseases, i.e. co-morbidities including diabetes and cardiovascular diseases [9–11].

Given the obesity-driven pathophysiology of MetS, the main driver for weight gain is considered to be the surplus of energy caused by excessive caloric intake (overnutrition) and/or combined with insufficient energy utilization, characterized by a sedentary lifestyle with little physical activity [1, 12]. Treatment of MetS is therefore often aimed at diminishing the surplus of energy in the system. This can be accomplished by making adjustments at both sides of the equation, but we are in particular interested in how increasing energy expenditure (EE) could contribute to the treatment of MetS.

Energy expenditure comprises multiple entities that consume energy, of which the most important ones include basal metabolic activity to maintain e.g. body temperature, and skeletal muscle activity. The latter can easily be stimulated by increasing physical activity. However, brown adipose tissue (BAT) also plays an important role in thermogenesis and energy management [13–17]. Recent studies have shown that activation of BAT has beneficial effects on weight loss, implying that this may be a promising therapeutic target against MetS [18–20]. Activated BAT combusts substantial amounts of triglycerides and glucose in the circulation [20–24]. A clinically feasible way to activate BAT is by cold exposure [25, 26]. Most of these studies do show an increased energy expenditure, but allow for direct compensation by increased food intake. To aid our understanding, we demonstrate a method to study the effects of increased energy expenditure isolated from other possible compensatory mechanisms. Since the effectiveness of such treatments may also strongly depend on the differential response of patients, our method will also take this into consideration.

Previously, we have developed a computational modelling framework describing the progressive and heterogeneous development of MetS [27]. This study yielded an extensive library of *N* = 1000 different model realizations. This ensemble of models was established by Monte Carlo sampling of experimental data assessed from a pre-clinical mouse model that describe onset and development of diet-induced MetS over a timespan of 3 months. This Monte Carlo sampling entails the generation of random samples of the data to account for experimental uncertainties. Subsequent model fitting yielded alternative parameterizations that describe the same phenotypic readout (in terms of plasma and liver biomarkers characteristic for MetS), but are established by different combinations of underlying model parameters and metabolic fluxes to match the sampled data to which this model realization was calibrated.

This collection of *n* = 1000 model realizations entails uniquely different parameter sets and different model outcomes. However, since the data to which each model instance has been calibrated was sampled from the same experimental data set, these different model realizations do describe the same overall observable phenotype. Each model realization yields a different model outcome, which is a result of quantification of uncertainty that was introduced by variability in data. So far, this collection of models was analyzed on the population level. Here, we evaluate this virtual patient cohort using an individualized perspective using so-called virtual patients [28–32]. Virtual patients can be regarded as different sets of model simulations that are representative of the differences in real-life. These virtual patients can subsequently be used in virtual trials to delineate how different individuals may respond differently to perturbations to the system and hence how effective potential treatment interventions may be [33].

Whereas food intake was explicitly incorporated in the model, the energy balance had not been analyzed. To identify differences between virtual patients in terms of energy handling, we first quantify the variation in energy expenditure and resulting energy balance and use this information for further stratification. Since the virtual patient cohort consists of *N* = 1000 different model realizations, we expect to find various combinations of metabolic fluxes underlying MetS.

Secondly, we analyze how robust the system is to changes in energy handling. Sensitivity and control of this type of metabolic systems are often assessed by applying perturbation experiments and is similar to methodologies often used in metabolic control analysis and flux balance analysis [34, 35]. Here we apply perturbations that induce increased peripheral energy expenditure, representing an increase in BAT activity. Energy is expended in the model by both the liver and the periphery. Peripheral tissues include metabolically active tissues such as skeletal muscle and adipose tissue. Hence, peripheral energy expenditure describes, amongst others, thermogenesis by BAT [13, 16, 36]. We therefore hypothesize that by simulating an increase in peripheral energy expenditure, activation of BAT can be studied in an in silico setting. We expect this additional drainage of energy from the peripheral compartment to diminish the energy surplus in the system. We hypothesize this perturbation leads to a decrease in peripheral triglyceride pool and also result in improvement in plasma biomarkers.

## Results

### Computational model of energy management in metabolic syndrome

**Fig. 1:**
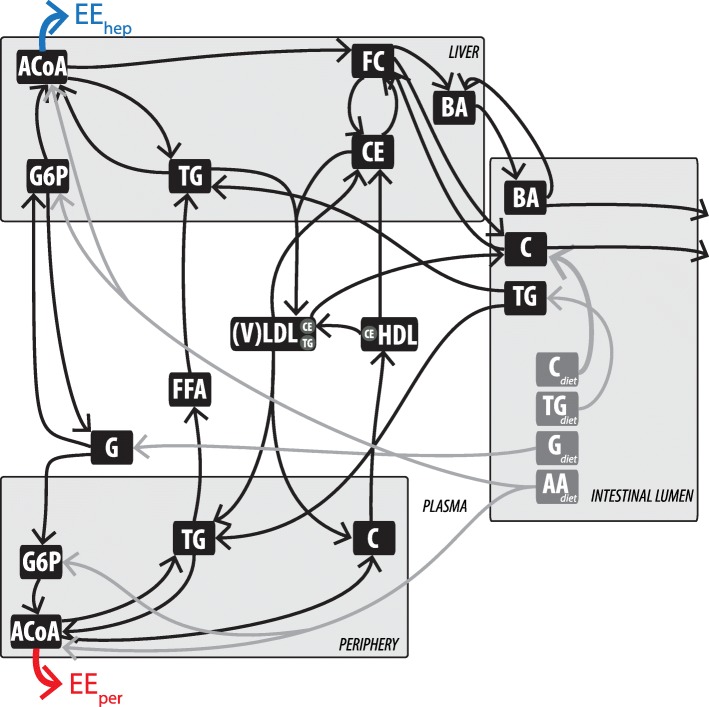
Schematic overview of energy expenditure in the computational model MINGLeD. Energy expenditure takes place in both hepatic (indicated by the blue arrow) and peripheral (indicated by the red arrow) compartments. This model scheme was adapted with permission from [27]. This multi-compartment framework encompasses pathways in dietary absorption, hepatic, peripheral, and intestinal lipid metabolism, hepatic, and plasma lipoprotein metabolism and plasma, hepatic, and peripheral carbohydrate metabolism. The metabolite pools in the different tissue compartments are displayed in the black frames; the corresponding metabolic fluxes are represented using the arrows. The grey fluxes represent the dietary inflow in terms of the different macronutrients derived from the experimental data. AA, amino acid; ACAT, Acyl-coenzyme A:cholesterol acyltransferase; ACoA, Acetyl CoA; BA, bile acid; C, cholesterol; CE, cholesteryl ester; CEH, cholesterol ester hydrolase; CETP, cholesteryl ester transfer protein; CM, chylomicron; DNL, de novo lipogenesis; (F)C, (free) cholesterol; (F)FA, (free) fatty acid; G, glucose; G6P, glucose-6-phosphate; GNG, gluconeogenesis; HDL, high density lipoprotein; TG, triglyceride; TICE, transintestinal cholesterol absorption; (V)LDL, (very) low density lipoprotein

The previously published computational model describing the metabolic system in both healthy and Metabolic Syndrome conditions (Model Integrating Glucose and Lipid Dynamics; MINGLeD) [27] is schematically displayed in Fig. 1. MINGLeD consists of four compartments (liver, intestine, plasma and periphery) in which carbohydrate, lipid, and cholesterol species are described. The peripheral compartment comprises the major metabolic tissues (except for the liver, intestine, and plasma) including adipose tissue and (skeletal) muscle.

MINGLeD describes energy handling with two components: energy intake (known from food intake data; depicted by the grey fluxes from the intestinal lumen) and energy expenditure (EE; predicted by the model). Energy expenditure is represented by respiration of acetyl-Co enzyme A (ACoA) in the liver (indicated by the blue arrow; EE_hep_) and in the peripheral compartment (indicated by the red arrow; EE_per_).

The model was previously calibrated to data derived from APOE3L.CETP mice which respond in a human-like manner [37, 38] to a high-fat diet supplemented with cholesterol, thereby inducing MetS. This data set [27] comprises monthly samples of plasma metabolite pool sizes and body weight and composition over the course of 3 months and was used for calibration of the model using maximum likelihood estimation. Here we will specifically utilize the *N* = 1000 model realizations subset representing the onset and progression dyslipidemic MetS [27]. This phenotype presents itself with the development of obesity and glucose intolerance in combination with dyslipidemia (high levels of plasma total cholesterol and high levels of plasma triglycerides). The collection of model realizations comprises of trajectories (model simulations over a timespan of 3 months) describing the metabolic pool sizes and fluxes in the plasma, liver, intestine, and periphery.

### Stratification of energy expenditure

Prior to applying any constraints on energy handling, any individuals that did not comply with the calibration data – i.e. did not accurately describe the data on which the trajectories were constraint – or those with unrealistic (high) flux magnitudes were excluded. This yielded a collection of *N* = 887, i.e. virtual individuals with physiologically correct MetS biomarkers.

**Fig. 2:**
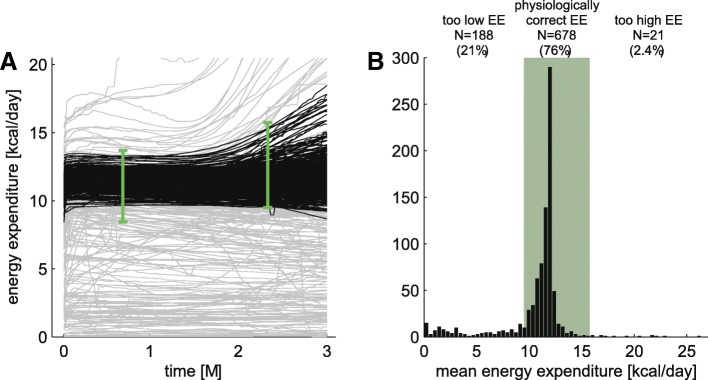
Energy expenditure predicted by MINGLeD as trajectories over time (**a**) and mean over time (**b**). **a** distribution of trajectories describing total energy expenditure. The trajectories that adhere to the physiological constraints (represented by the green error bars; see Table 1) are depicted in black; the unacceptable ones in grey. **b** histogram of the mean energy expenditure. The physiologically acceptable range is depicted in green and derived from the following inclusion criteria: -EE at t = 3w within three-weeks confidence interval, i.e. [8.4–13.7 kcal/day]; −EE at t = 10w within three-weeks confidence interval, i.e. [9.5–15.7 kcal/day]; −overall minimum EE above the lower bound of the 3w confidence interval, i.e. 8.4 kcal/day; −overall maximum EE below 20 kcal/day

However, the models should not only adequately describe biomarkers, but energy handling is also an important criterion for model selection. While energy intake is known from food intake, energy expenditure is not yet studied. Therefore, we first stratify the population to ensure physiologically plausible values of energy expenditure in the system. Figure 2a shows the distribution of trajectories of total energy expenditure (summation of hepatic and peripheral EE) over time. The timespan on the horizontal axis describes development from a healthy phenotype to MetS over a period of 3 months. The collection of trajectories contains models ranging from low to high energy expenditure, but in general, the EE remains relatively stable over time. Therefore, the mean, as shown in the histogram of Fig. 2b, is sufficient to summarize these results.

We applied physiological constraints obtained via indirect calorimetry (see Table 1 in the Methods section). Metabolic cages were used to measure VO_2_ and VCO_2_ such that metabolic rate and energy expenditure can be quantified [39, 40]. The physiological constraints obtained from these experiments aredepicted as the 99.7% confidence interval (green error bars in Fig. 2a and green shaded area in Fig. 2b). This demonstrates that the majority of the virtual population (76%; *N* = 678) presented itself with a physiologically plausible energy expenditure. Models with extremely low EE and high EE are presumed to be artefacts of solving the inverse problem of fitting a model with many degrees of freedom to a limited amount of data.

**Table 1:** Total energy expenditure assessed using indirect calorimetry

	EE [kcal/day]	fat oxidation [%]	carbohydrate oxidation [%]	protein oxidation [%]
MetS-3w	11.1 ± 0.87 [8.4–13.7]	57.6 ± 2.8 [49.3–65.9]	22.4 ± 2.8 [14.1–30.7]	20.0 ± 2.6e-4 [20.0–20.0]
MetS-10w	12.6 ± 1.04 [9.5–15.7]	63.2 ± 2.0 [57.4–69.1]	16.8 ± 2.0 [10.9–22.6]	20.0 ± 1.3e-4 [20.0–20.0]

Data is depicted as mean ± standard deviation, and as 99.7% confidence interval (between brackets)

For the following analyses we limit ourselves to the subgroup of *N* = 678 virtual individuals. With an average energy expenditure of 12 kcal/day (calculated by the model) and an average energy intake of 19 kcal/day (known from dietary composition and daily food intake), the resulting energy balance is a constant surplus of energy of around 7 kcal/day. This explains the weight gain and development of obesity over time.

### Energy is mainly expended in the peripheral compartment

**Fig. 3:**
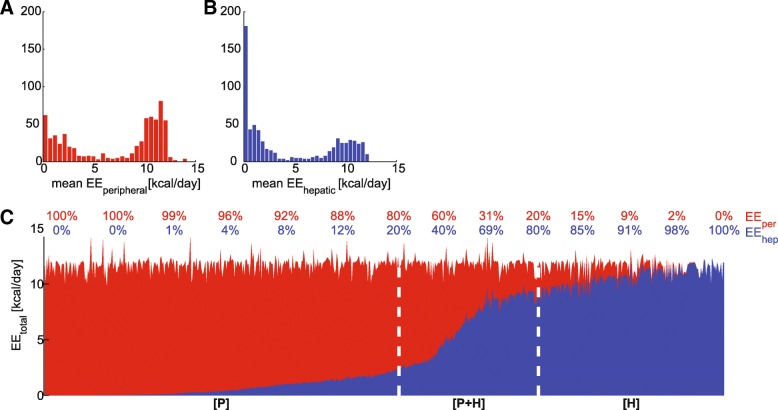
Peripheral (red) and hepatic (blue) contribution of energy expenditure. **a** and **b** include histograms of the mean energy expenditure. **c** shows the relative contribution (numbers above graph) where each vertical line represents a single virtual individual. The division in subgroups [P], [P + H], and [H] is indicated by the white dashed lines

The next step in the stratification process comprises the breakdown of the contribution of different tissues to the total energy expenditure. The total energy expenditure consists of energy utilization in both liver (Fig. 3a) and periphery (Fig. 3b). We expected to find a significant contribution from the periphery, compared to the liver. The periphery is the largest compartment (both in volume and in the number of cells). Since it also comprises muscle and BAT, we expect that the periphery utilizes much more energy than the liver, although the liver is also a metabolically active organ. However, we found a distribution with a strong bimodal profile. This bimodality indicates that energy can predominantly be utilized by just either of these tissues, but that energy can also be utilized by both compartments to the same extent.

Consequently, we divided the population into three different subgroups, each with its own characteristic contribution of hepatic and peripheral energy expenditure. Figure 3 c shows the relative contribution of hepatic (blue) and peripheral (red) EE for each virtual individual at the three-month’s time point of MetS development. It reveals the existence of a continuous “spectrum” in the contribution of hepatic and peripheral energy expenditure. As suggested by Fig. 3c, in a part of the population, the majority of energy is utilized in the periphery (on the left-hand side); another subgroup exists in which the majority of energy is utilized in the liver (on the right-hand side); and an intermediate group in which both peripheral and hepatic energy expenditure are significantly contributing to the total energy consumption. Therefore the virtual individuals were separated into three different subgroups:

- [P]: predominantly peripheral energy expenditure (> 80% originates from the peripheral compartment);
- [H]: predominantly hepatic energy expenditure (> 80% originates from the hepatic compartment);
- [P + H]: intermediate subgroup in which both periphery and liver contribute significantly (> 20% originates from the peripheral compartment and > 20% originates from the liver).

Additional file 1: Table S1 lists the characteristics for each of these subgroups, and shows that these subgroups are clearly separated in their average peripheral and hepatic energy consumption. Additional file 2: Figure S1 shows that although the predominant compartment of energy expenditure varies among these individuals, the same MetS phenotype in terms of biomarker profiles has been established, whereas the underlying metabolic fluxes may be different (see Additional file 2: Figure S2).

### Further stratification based on substrate oxidation

The subsequent step of the stratification process involves further specification of the source of energy. Energy expenditure in MINGLeD is described by the respiration of ACoA. The ACoA pool originates from three different substrates: carbohydrate, lipid, and protein. ACoA is obtained from carbohydrate substrates via glycolysis of glucose-6-phosphate. ACoA from lipid substrate originates from the β-oxidation of triglycerides. ACoA can also be derived from ketogenic protein uptake from the diet.

**Fig. 4:**
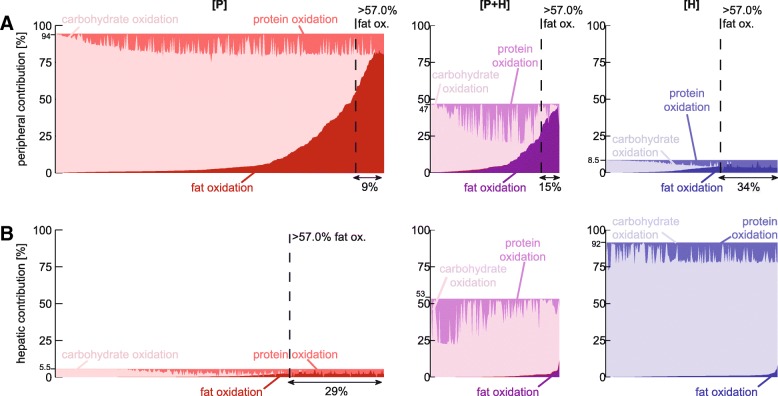
Contribution of carbohydrate and fat oxidation to the peripheral (**a**) and hepatic (**b**) energy expenditure. In subgroup [P] (left-hand side panels), energy is predominantly utilized in the periphery (> 80% originates from the peripheral compartment). In subgroup [H] (right-hand side panels), energy is predominantly utilized in the liver (> 80% originates from the hepatic compartment). Subgroup [P + H] (panels in the center) is an intermediate subgroup in which both periphery and liver contribute significantly (> 20% originates from peripheral compartment and > 20% originates from the liver). The dark colored areas (bottom right) correspond with fat oxidation, the medium colored areas (top left) indicate protein oxidation and the light areas (middle) specify carbohydrate oxidation. The dashed line bounds of the acceptable physiological range on the lipid oxidation ratio (at least 57% originates from lipid substrates). The fraction of individuals that adheres to this constraint is depicted below each graph

In Fig. 4 the relative peripheral (A) and hepatic (B) energy utilization are shown, split up into the relative contribution of carbohydrate, lipid, and protein oxidation. MINGLeD predicts a range of substrate ratios (carbohydrate:lipid:protein) to be possible and predicts that the majority of the virtual individuals utilize mainly carbohydrate substrates as an energy source while lipids are stored in the form of triglycerides (TG).

Literature has revealed that on a high-fat diet, mammals mainly utilize TG as energy source [41–44]. In our diet-induced MetS animal model, physiological data (see Table 1 in the Methods section) has placed this cut-off on at least 57% of energy to result from lipid substrates. Therefore we imposed this as criterion for the minimal contribution of fat oxidation, indicated by the dashed line in Fig. 4.

Additional file 3: Table S2 lists the overall statistics of the substrate oxidation for peripheral and hepatic energy expenditure for each subgroup separately. In the predominantly peripheral subgroup [P], overall, 75% of peripheral energy originates from carbohydrate sources, 15% from fat oxidation and 10% from protein substrates. Although most of these numbers are not close to our 57% fat-threshold, a subset of this group does adhere to this criterion (highlighted in grey in Additional file 3: Table S2).

Note that in the predominantly hepatic subgroup [H], only acceptable solutions regarding the relative contribution of fat oxidation were found for the peripheral energy consumption, but that the contribution of the periphery to the total energy expenditure is very low (< 10%).

To conclude, our stratification process resulted in a reduced population of *N* = 32 virtual individuals. This is a representative subgroup as the selected individuals 1) have an accurate description of plasma and liver biomarkers (the characteristic MetS phenotype), 2) have a physiologically correct EE, 3) predominantly utilize energy in the periphery, and 4) of which energy originates for at least 57% from lipid substrates.

This stratification and selection process reduced the virtual population of interest from several hundred to a few dozen virtual individuals. Since each virtual individual in the selected subgroup is described by a different parameter set, we decided to analyze each model in more detail to understand how differences in model parameters affect the behavior of the metabolic system. For this analysis, the subgroup of *N* = 32 virtual individuals was sufficiently large to represent the variability within the population and to interpret results on an individual basis.

### In silico perturbation experiment to study the robustness of energy homeostasis

**Fig. 5:**
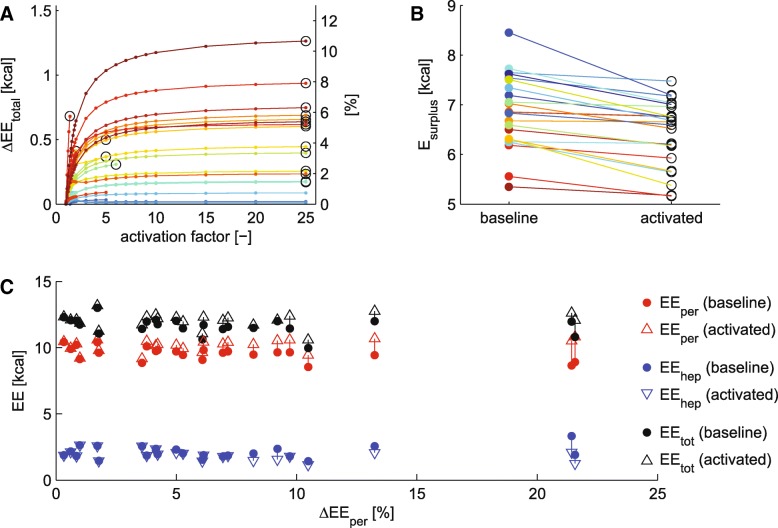
In silico activation of peripheral energy expenditure leads to an increase of total EE. **a** shows the absolute (left vertical axis) and relative (right vertical axis) change in total EE upon increasing activation factor. Each line depicts a different virtual individual where data are color-coded according to the maximally achieved increase in peripheral energy expenditure. For each virtual individual, the highest activation result (if yielding at least 0.1% increase in total EE) was used for further analysis and indicated by the black circle. **b** displays the resulting decrease of the energy surplus in the system. Results are color-coded based on (**a**). **c** presents the shift in peripheral (red), hepatic (blue) and total (black) EE from baseline (represented with dots) to in silico activation (represented with upward facing triangles for increasing values and downward triangles for decreasing values) versus the relative increase in peripheral EE (on the horizontal axis)

Subsequently, we employed MINGLeD to simulate an increase in peripheral energy expenditure. To induce an increase in peripheral energy expenditure, we perturbed each of the *N* = 32 selected models (that adhered to physiological constraints in terms of EE and substrate oxidation) by multiplication of the peripheral ACoA flux with different activation factors as shown in Fig. 5a. This factor was iteratively increased from 1 to 25 as explained in detail in the Methods section. For each factor, the steady-state of the model system was re-calculated while the nutritional intake was kept constant at the original values for macronutrient intake. Since each virtual individual is described by a different parameter set, the different individuals can be expected to respond differently to perturbation in energy balance.

Figure 5a demonstrates the effects of perturbation in energy expenditure (results are color-coded for each model) versus the activation factor on the horizontal axis. Whereas these models respond differently to the perturbation, the majority shows a strong increase in total energy expenditure upon increasing values of the activation factor and saturating towards a plateau. However, the level of the plateau is different throughout our population. This means that in some individuals, the peripheral energy expenditure can be activated to a much larger extent than for others. For further analysis, we selected the activation factor that achieved highest increase in EE (indicated by the black circle), yielding at least an increase of 0.1% in total energy expenditure – as an increase in total energy expenditure should be substantial in order to induce propagation of effects throughout the system. This showed that the perturbation was successfully applied in 23 virtual individuals.

The maximally achieved increase in EE is different for each individual and for some the effects of the perturbation are much higher than for others (Fig. 5a). For some models, application of larger activation factors led to depletion of the peripheral ACoA pool, preventing a further increase in the externally applied perturbation (these are the solutions that do not span the entire horizontal axis).

The perturbation yielded a decrease of the energy surplus (Fig. 5b) of up to 2 kcal, but not sufficient to create an energy deficit. Under the condition of fixed food intake, increase in peripheral EE (Fig. 5c in red) is paralleled by a decrease in hepatic EE (Fig. 5c in blue). This decrease in hepatic EE is more profound when the increase in peripheral energy expenditure is higher (towards the right on the horizontal axis) – but the total EE (Fig. 5c in black) does increase upon increased peripheral EE.

**Fig. 6:**
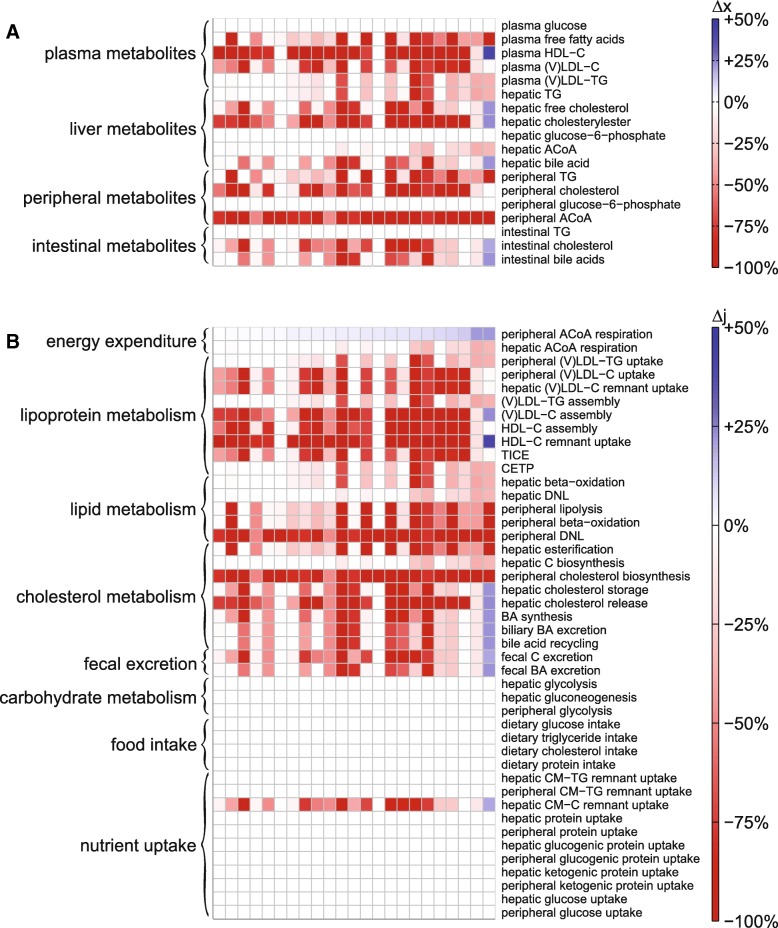
Increased peripheral energy expenditure affects metabolite pools (**a**) and metabolic fluxes (**b**) throughout the system. The impact of the activation is depicted as relative change using a heatmap for *N* = 23 virtual individuals (from left to right: increasing relative change of peripheral energy expenditure). The changes are color-coded such that decreases are shown in red and increases in blue, and according to intensity: a darker color indicates a stronger change in metabolite concentration than a lighter color. White indicates 0% change

Figure 6 shows the resulting relative change in metabolite pool sizes (A) and metabolic fluxes (B) upon the highest achieved increase in total EE. Using heatmaps, we depicted these results for the *N* = 23 different individualized models with from left to right increasing relative change of peripheral EE. Decreasing pool sizes and fluxes are shown in red and increases in blue.

Perturbation induced a drastic increase in peripheral ACoA respiration (top row in Fig. 6b), obviously depleting large quantities of the peripheral ACoA pool. Results reveal direct changes in peripheral lipid and lipoprotein metabolism, but also propagation into the plasma, liver, and intestine. Circulating lipoprotein levels decrease with increased peripheral energy expenditure (whilst dietary intake was kept the same). Remarkably, the perturbation did not imply any changes in the carbohydrate metabolic system.

## Discussion

We successfully studied energy handling in metabolic syndrome development. Our perturbation experiments have shown that an additional drain of peripheral energy expenditure successfully decreases lipid and lipoprotein pools in the periphery, but also lipid contents in the surrounding tissues. This thereby provided insight into how a change in energy handling could be beneficial in the treatment of MetS.

The growing incidence rates of obesity and related diseases in combination with the heterogeneity in phenotypic presentation and metabolic manifestations ask for a more patient-specific approach towards treatment [45, 46]. Hereto we should first gain insight in which patient subgroups can be identified. Recently we have demonstrated the differential response to high-fat, high-cholesterol feeding, which induces two different MetS phenotypes [27]. These findings are in line with the expected phenotypic heterogeneity in metabolic component combinations [47, 48], but also the heterogeneity within the same phenotypic presentation – and energy handling – may be large. Whereas most conventional studies make predictions based on the population level, we therefore took a step further and evaluated virtual patient subgroups. This follows the path towards evaluating individual, patient-specific data and enabling predictions in an individualized framework by classifying patients into a corresponding subgroup [32].

We have shown the feasibility of (virtual) patient stratification. Relevant individuals were first filtered out based on physiological constraints. This approach parallels with many in vivo experimental setups as a reduction of the population is applied to retain only those individuals expressing the desired features, but also yielding a manageable amount of data. This is a crucial step in the “era of precision medicine” [49] towards identifying a framework to classify patients in corresponding subgroups and often used in virtual (and clinical) trials [50, 51].

Our perturbation experiment can also be regarded as a virtual trial. For this we even took one step further and provided simulations on an individual level. For instance, we can demonstrate that enhanced peripheral energy expenditure can be used as an in silico proxy to study the effects of BAT activation. Firstly, the imposed perturbation is in the same order of magnitude as achieved in clinical practice with exposure to cold (despite possibly extra energy intake). In our virtual individuals, large differences have been observed to what extent the energy expenditure could be increased. However, (pre)clinical studies report that cold exposure (CEX) induces a similarly large range of average increase of energy expenditure compared to thermo-neutral conditions ranging from only a few percent to several dozen percents increase [25, 52, 53]. These results strongly depend on the conditions of the experiment: degree (mild versus strong, i.e. how cold) and the duration of the period of cold exposure.

Secondly, the imposed flux changes are in line with clinical observations showing that BAT can be activated by cold exposure [25, 26, 54–57] and that it possesses anti-obesogenic properties [52]. We found a reduction of (circulating) lipid and cholesterol levels after simulating short and acute CEX. Radioactive tracer experiments confirmed direct changes in TG uptake fluxes after one-day of CEX but did not report changes in plasma markers [21, 58].

This difference could be explained by differences in experimental conditions: the in silico study induced a quite extreme activation compared to one-day CEX treatment. If CEX would have induced an activation as strong as in the in silico case, supposedly changes in plasma metabolite pools would have been observed as well.

Literature indicates that BAT also possesses the ability to improve glucose handling. This is in contrast to our results as the carbohydrate system remains unaffected upon increased peripheral EE. This difference may be explained by considering the model’s stoichiometry, and more specifically, the direction of the fluxes in the model. MINGLeD was designed to describe the most important elements and processes in lipid, cholesterol, and carbohydrate metabolism. Thorough model testing using different scenarios or metabolic conditions (such as in this study the in silico activation of peripheral EE) indicates how MINGLeD can be extended and improved. The observation that the glucose system remains unaffected upon increased peripheral EE is an indication to further investigate the relationship between glucose and ACoA, which is currently implemented as a one-way interaction (glucose-6-phosphate can only be donated to ACoA).

Experimental studies have shown that BAT activation can induce weight loss [18–20]. Our perturbation experiment has shown that increased peripheral energy expenditure is able to induce a decrease of the energy surplus in the system. To yield a negative energy balance, we would recommend longer and/or more frequent periods of CEX treatment to induce a sustained and/or prolonged BAT activation. Experimental studies with intermittent CEX schemes have shown to be feasible to do this [22, 59, 60]. Recent studies have also shown the potential to chronically activate BAT using a pharmacological intervention with the thermogenic β3-adrenergic receptor agonist CL316,243 [23, 61]. Dietary supplementation of the short-chain fatty acid butyrate has shown promising results in both animals [62] and in humans [63, 64] to reduce both appetite and active BAT through the gut-brain axis.

Most computational models describing energy metabolism are specifically developed for the human metabolic system, and hardly any for murine energy metabolism [65]. Whereas specific metabolic pathways may be different between mouse and human [66], much can be learnt from mouse computational models. Our computational model (Fig. 1) was designed to be a generic representation for both murine and human energy metabolism. Since no human data is available as of yet, our work using the murine model calibration provides a step towards translation of in silico models developed using genetically modified mice towards the human energy management in metabolic diseases.

## Conclusions

The computational analysis of energy handling and energy expenditure for stratification and perturbation experiments confirmed that the energy imbalance plays an important role in the development of obesity and its related diseases. Furthermore, increasing peripheral energy expenditure has a positive effect on lipid metabolism in Metabolic Syndrome.

## Methods

### Stratification of energy handling in an in silico model

We employ our previously developed computational model MINGLeD (Model Integrating Glucose and Lipid Dynamics) describing the metabolic system from a healthy state towards development to Metabolic Syndrome [27]. MINGLeD is composed of ordinary differential equations that have been implemented in MATLAB (2013b, The Mathworks, Natick, Massachusetts), which is available on GitHub (via github.com/yvonnerozendaal/MINGLeD).

MINGLeD was utilized in combination with ADAPT (Analysis of Dynamic Adaptations in Parameter Trajectories) [67–69] to achieve a model library describing various phenotypes. Here we analyze the *N* = 1000 model simulations for the dyslipidemic Metabolic Syndrome phenotype. These model simulations describe MetS development over a timescale of 3 months, with a discretization of 90 days. Based on this large set of in silico data, we performed data reduction by applying physiological constraints to obtain a manageable amount of physiologically-correct data.

Physiological data on the energy expenditure was obtained using metabolic cages (see also subsequent Methods paragraphs). In the experimental study of [27], the animals were subjected to indirect calorimetry after 3 and 10 weeks of diet induction. This information was used to select those virtual individuals of which the energy expenditure lies within a physiologically correct range, defined using both the three-week (8.4–13.7 kcal/day) and the 10-week (9.5–15.7 kcal/day) 99.7% confidence interval (see Table 1).

Moreover, this physiological data was also utilized to define a threshold for the relative contribution of fat oxidation to energy expenditure. As criterion we used that for mice on a high-fat diet at least 57% of the energy should originate from lipid substrates. This cut-off value is based on the lower bound of the 99.7% confidence interval for fat oxidation (see Table 1) obtained by indirect calorimetry after 10 weeks of MetS induction (since this resembles the fully developed phenotype the best). The virtual individuals we selected for further analysis predominantly utilize energy in the periphery (subgroup [P]), with approximately 75% of energy from carbohydrates, 15% from fat oxidation and ~ 10% from protein, resulting in a cohort of *N* = 32 individuals that was used for further analysis.

### Converting energy expenditure into energy units

Traditionally, all fluxes in MINGLeD are expressed in μmol/day. To recalculate the energy expenditure fluxes in MINGLeD into energy units, we made use of the energy content of TG particles. Hereto, we first recalculated the ACoA respiratory fluxes into the equivalent of TG particles assuming that 1 mol of TG is equivalent to 21.4 mol of ACoA:

1 $$ EE\left[\frac{\upmu \mathrm{mol}\ \mathrm{TG}}{\mathrm{day}}\right]= EE\left[\frac{\upmu \mathrm{mol}\ \mathrm{ACoA}}{\mathrm{day}}\right]\cdot \frac{1}{21.4} $$

Then these fluxes were converted from molar units to grams per day by assuming that the molar mass of TG is 853 u:

2 $$ EE\left[\frac{\mathrm{g}\ \mathrm{TG}}{\mathrm{day}}\right]= EE\left[\frac{\upmu \mathrm{mol}\ \mathrm{TG}}{\mathrm{day}}\right]\cdot 853\cdot {10}^{-6}\left[\frac{\mathrm{g}}{\upmu \mathrm{mol}}\right] $$

And then we can calculate how much energy is equivalent to this flux assuming that 1 g of fat contains 9 kcal:

3 $$ EE\left[\frac{\mathrm{kcal}}{\mathrm{day}}\right]= EE\left[\frac{\mathrm{g}\ \mathrm{TG}}{\mathrm{day}}\right]\cdot 9\left[\frac{\mathrm{kcal}}{\mathrm{g}}\right] $$

Hence, the energy expenditure fluxes can easily be converted from molar units into energy content using:

4 $$ EE\left[\frac{\mathrm{kcal}}{\mathrm{day}}\right]= EE\left[\frac{\upmu \mathrm{mol}\ \mathrm{ACoA}}{\mathrm{day}}\right]\cdot \frac{1}{21.4}\cdot 853\cdot {10}^{-6}\cdot 9 $$

### Physiological ranges provided by in vivo assessment of energy expenditure

Male E3L.CETP transgenic mice (as described in [27]) were housed in a temperature-controlled environment (21 °C) under standard conditions with a 12 h light/dark cycle (7 AM-7 PM), with free access to diet and water in individually ventilated cages, unless indicated otherwise. At the age of 11 weeks, mice were fed a high-fat, high-cholesterol diet (the same individuals as were the subjects in the previously published study [27]) for 3 months. To measure energy expenditure in the in vivo situation, after 3 and 10 weeks of diet induction, respectively, mice (*n* = 8) underwent indirect calorimetry using metabolic cages. Mice were housed individually in these metabolic cages for 4 days. The first day is used to let the mice get used to the new environment. The animals were non-invasively, fully computer operated monitored during these 4 days. Afterwards the animals were put back into their normal cages.

O_2_ and CO_2_ concentrations were measured every 10 min to calculate the energy expenditure [40]. Different substrates yield different consumption rates. We can infer the relative contribution of substrate utilization from the measured changes in oxygen and carbon dioxide:

5 $$ {\displaystyle \begin{array}{c}{EE}_{total}={EE}_{glucose}+{EE}_{fat}+{EE}_{protein}\\ {}{EE}_{glucose}={VO}_2\cdot {f}_{glucose}\cdot {RED}_{glucose}\\ {}{EE}_{fat}={VO}_2\cdot {f}_{fat}\cdot {RED}_{fat}\\ {}{EE}_{protein}={VO}_2\cdot {f}_{protein}\cdot {RED}_{protein}\end{array}} $$

where *VO*_*2*_ represents consumed oxygen (L O_2_/day), *RED*_*x*_ is the respiratory energy density (in kcal/L O_2_) of substrate *x* and *f*_*x*_ is the relative contribution to the total oxygen consumption by oxidation of substrate *x*. Based on the respiratory quotient (RQ):

$$ RQ=\frac{VCO_2}{VO_2} $$

6 $$ RQ={f}_{glucose}\bullet {RQ}_{glucose}+{f}_{fat}\bullet {RQ}_{fat}+{f}_{protein}\bullet {RQ}_{protein} $$

assuming *RQ*_*glucose*_ = 1, *RQ*_*fat*_ = 0.71, *RQ*_*protein*_ = 0.835 [70], the respiratory energy density parameter should adhere to:

7 $$ {f}_{glucose}+{f}_{fat}+{f}_{protein}=1 $$

Assuming that body mass of protein is constant, the rate of protein oxidation should equal the rate of protein intake. Hence, protein oxidation will be a consistent factor *γ* of the total energy expenditure:

8 $$ {EE}_{protein}=\gamma \bullet \left({EE}_{glucose}+{EE}_{fat}+{EE}_{protein}\right) $$

Substitution with Eq. (5) and some rearranging yields:

9 $$ {f}_{protein}=\frac{\frac{\gamma }{1-\gamma}\bullet \left({f}_{glucose}\bullet RE{D}_{glucose}+{f}_{fat}\bullet RE{D}_{fat}\right)}{RED_{protein}} $$

which can be simplified using substitution with α and β by:

10 $$ {\displaystyle \begin{array}{c}\alpha =\frac{\gamma }{1-\gamma}\cdot \frac{RED_{glucose}}{RED_{protein}}\\ {}\beta =\frac{\gamma }{1-\gamma}\cdot \frac{RED_{fat}}{RED_{protein}}\end{array}} $$

and yields:

11 $$ {f}_{protein}=\alpha \bullet {f}_{glucose}+\beta \bullet {f}_{fat} $$

Therefore the relative contribution of the other substrates is determined by:

12 $$ {\displaystyle \begin{array}{c}{f}_{glucose}=\frac{RQ-\frac{\left({RQ}_{fat}+\beta \cdot {RQ}_{protein}\right)}{1+\beta }}{RQ_{glucose}+\alpha \cdot {RQ}_{protein}-\frac{\left(1+\alpha \right)\left({RQ}_{fat}+\beta \cdot {RQ}_{protein}\right)}{1+\beta }}\\ {}{f}_{fat}=\frac{1-{f}_{glucose}\cdot \left(1+\alpha \right)}{1+\alpha}\end{array}} $$

assuming that *γ* = 0.2, *RED*_*protein*_ = 4.17, *RED*_*fat*_ = 4.66 and *RED*_*glucose*_ = 5.02 [70].

These statistics of the obtained calculations for the energy expenditure for the different substrates are depicted in Table 1.

### In silico perturbation experiment inducing enhanced peripheral energy expenditure

Since we aim to study the effects of short-term BAT activation through cold exposure, we chose to perform our in silico simulation using the one-day metabolic snapshot obtained in the fully developed phenotype, i.e. after 3 months of MetS induction. This timescale is also consistent with the time window in which an in vivo cold exposure intervention would be applied.

The perturbation experiment involved applying an external perturbation such that an in silico increase in peripheral energy expenditure was achieved. Since the peripheral compartment comprises of all metabolically active tissues apart from the liver, plasma, and intestinal lumen, we assumed that the respiration of peripheral acetyl Coenzyme A (represented by the red arrow in Fig. 1) can be used as a proxy for BAT activation.

An increase in peripheral ACoA respiratory flux was induced by multiplication of the flux equation with activation factor *f*_*act*_:

13 $$ {j}_{resp, per}^{ACoA}={k}_{resp, per}\cdot {ACoA}_{per}\cdot {f}_{act} $$

However, since it is not a priori known how high this activation factor should be, and this factor may differ among different virtual individuals, we applied a variety of activation factors that ranged different scales (1 + 1e-10, 1 + 1e-8, 1 + 1e-6, 1 + 1e-4, 1 + 1e-3, 1 + 1e-2, 1.1:0.1:1.9 2:9 10:5:25) to the system. The system was re-simulated to steady state with these perturbations applied, yielding the results presented in Figs. 5 and 6.

## Additional files

Additional file 1: **Table S1.** Division into subgroups characteristic for the peripheral and hepatic contribution to the total energy expenditure. (DOCX 15 kb)

Additional file 2: **Figure S1.** Metabolite pool sizes depend on where the majority of energy is utilized. The mean pool sizes in individuals with predominantly peripheral energy expenditure [P] are depicted in red; mean pool sizes in individuals with predominantly hepatic energy expenditure [H] in blue; and mean pool sizes of individuals with both peripheral and hepatic energy expenditure [P + H] in purple. All pool sizes of plasma metabolites are expressed as concentration in mM; all other pool sizes are expressed in μmol. **Figure S2.** Metabolic fluxes depend on where the majority of energy is consumed. The mean fluxes in individuals with predominantly peripheral energy expenditure [P] are depicted in red; mean fluxes in individuals with predominantly hepatic energy expenditure [H] in blue; and mean fluxes of individuals with both peripheral and hepatic energy expenditure [P + H] in purple. All fluxes are expressed in μmol/day. (DOCX 360 kb)

Additional file 3: **Table S2.** Relative contribution of substrate oxidation to peripheral and hepatic energy expenditure. The relative contribution of substrate oxidation is depicted as mean ± standard deviation, and the minimum and maximum bounds are denoted between brackets. The number of virtual individuals adhering to the physiological bound of at least 57% fat oxidation is highlighted in grey. (DOCX 16 kb)

## Abbreviations

[H]: Subgroup with mainly hepatic energy expenditure
[P + H]: Subgroup with both substantial hepatic and peripheral energy expenditure
[P]: Subgroup with mainly peripheral energy expenditure
ACoA: Acetyl Coenzyme A
ADAPT: Analysis of Dynamic Adaptations in Parameter Trajectories
BAT: Brown adipose tissue
CEX: Cold exposure
EE: Energy expenditure
EE_hep_: Hepatic energy expenditure
EE_per_: Peripheral energy expenditure
MetS: Metabolic syndrome
MINGLeD: Model Integrating Glucose and Lipid Dynamics
TG: Triglycerides
