# Supplementary material for: Computational modelling of energy balance in individuals with Metabolic Syndrome
Source: BMC Syst Biol. 2019 Feb 26;13:24. doi: 10.1186/s12918-019-0705-z (PMC6390597; doi:10.1186/s12918-019-0705-z)
Supplement: Supplementary file 1 — Table S1. Division into subgroups characteristic for the peripheral and hepatic contribution to the total energy expenditure. (DOCX 15 kb) [file 12918_2019_705_MOESM1_ESM.docx]

Additional file 1: Table S1: Division into subgroups characteristic for the peripheral and hepatic contribution to the total energy expenditure.

|  | N | relative contribution of peripheral EE [%] | | | | | relative contribution of hepatic EE [%] | | | | |
| --- | --- | --- | --- | --- | --- | --- | --- | --- | --- | --- | --- |
|  |  |  | mean | std | min | max |  | mean | std | min | max |
| [P] | 354 (52%) |  | 94 | 5.6 | 80 | 100 |  | 5.5 | 5.6 | 4.3e-7 | 20 |
| [P+H] | 139 (21%) |  | 47 | 21 | 20 | 80 |  | 53 | 21 | 20 | 80 |
| [H] | 185 (27%) |  | 8.5 | 6.5 | 2.6e-4 | 20 |  | 92 | 6.5 | 80 | 100 |
