# Supplementary material for: Computational modelling of energy balance in individuals with Metabolic Syndrome
Source: BMC Syst Biol. 2019 Feb 26;13:24. doi: 10.1186/s12918-019-0705-z (PMC6390597; doi:10.1186/s12918-019-0705-z)
Supplement: Supplementary file 2 — Figure S1. Metabolite pool sizes depend on where the majority of energy is utilized. The mean pool sizes in individuals with predominantly peripheral energy expenditure [P] are depicted in red; mean pool sizes in individuals with predominantly hepatic energy expenditure [H] in blue; and mean pool sizes of individuals with both peripheral and hepatic energy expenditure [P + H] in purple. All pool sizes of plasma metabolites are expressed as concentration in mM; all other pool sizes are expressed in μmol. Figure S2. Metabolic fluxes depend on where the majority of energy is consumed. The mean fluxes in individuals with predominantly peripheral energy expenditure [P] are depicted in red; mean fluxes in individuals with predominantly hepatic energy expenditure [H] in blue; and mean fluxes of individuals with both peripheral and hepatic energy expenditure [P + H] in purple. All fluxes are expressed in μmol/day. (DOCX 360 kb) [file 12918_2019_705_MOESM2_ESM.docx]

We identified three subgroups based on where the majority of energy is utilized: predominantly peripheral [P], predominantly hepatic [H] and both peripheral and hepatic energy expenditure [P+H]. Here we examined the metabolic fluxes and pool sizes corresponding to these different classes. Regardless of where the majority of energy is utilized, the resulting MetS phenotype in terms of plasma and liver biomarkers (see top rows of Figure S1) is equivalent. However, this phenotype can develop with different combinations of underlying metabolic fluxes (see Figure S2).


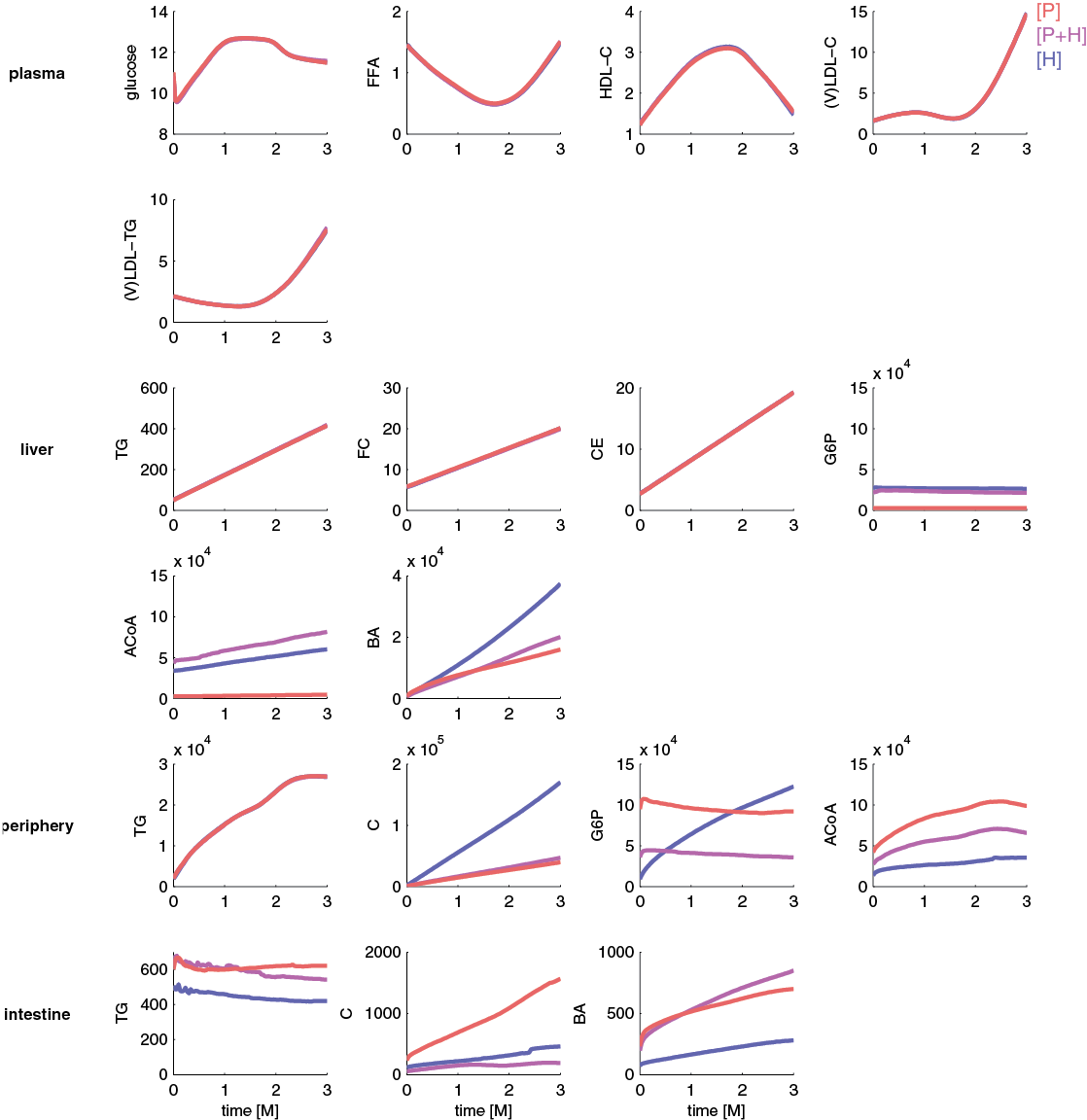


Additional file 2: Figure S1: Metabolite pool sizes depend on where the majority of energy is utilized.

The mean pool sizes in individuals with predominantly peripheral energy expenditure [P] are depicted in red; mean pool sizes in individuals with predominantly hepatic energy expenditure [H] in blue; and mean pool sizes of individuals with both peripheral and hepatic energy expenditure [P+H] in purple. All pool sizes of plasma metabolites are expressed as concentration in mM; all other pool sizes are expressed in µmol.


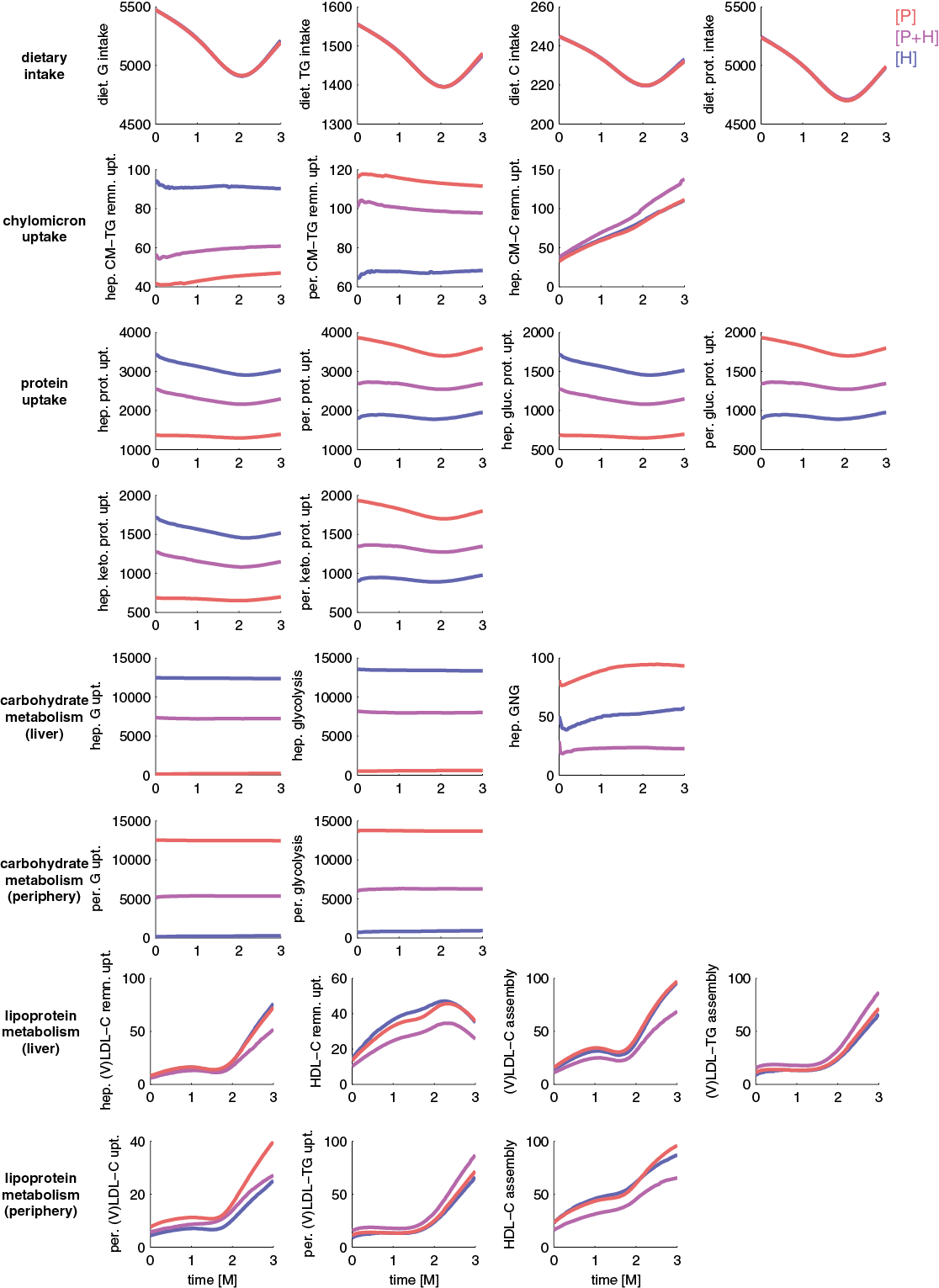


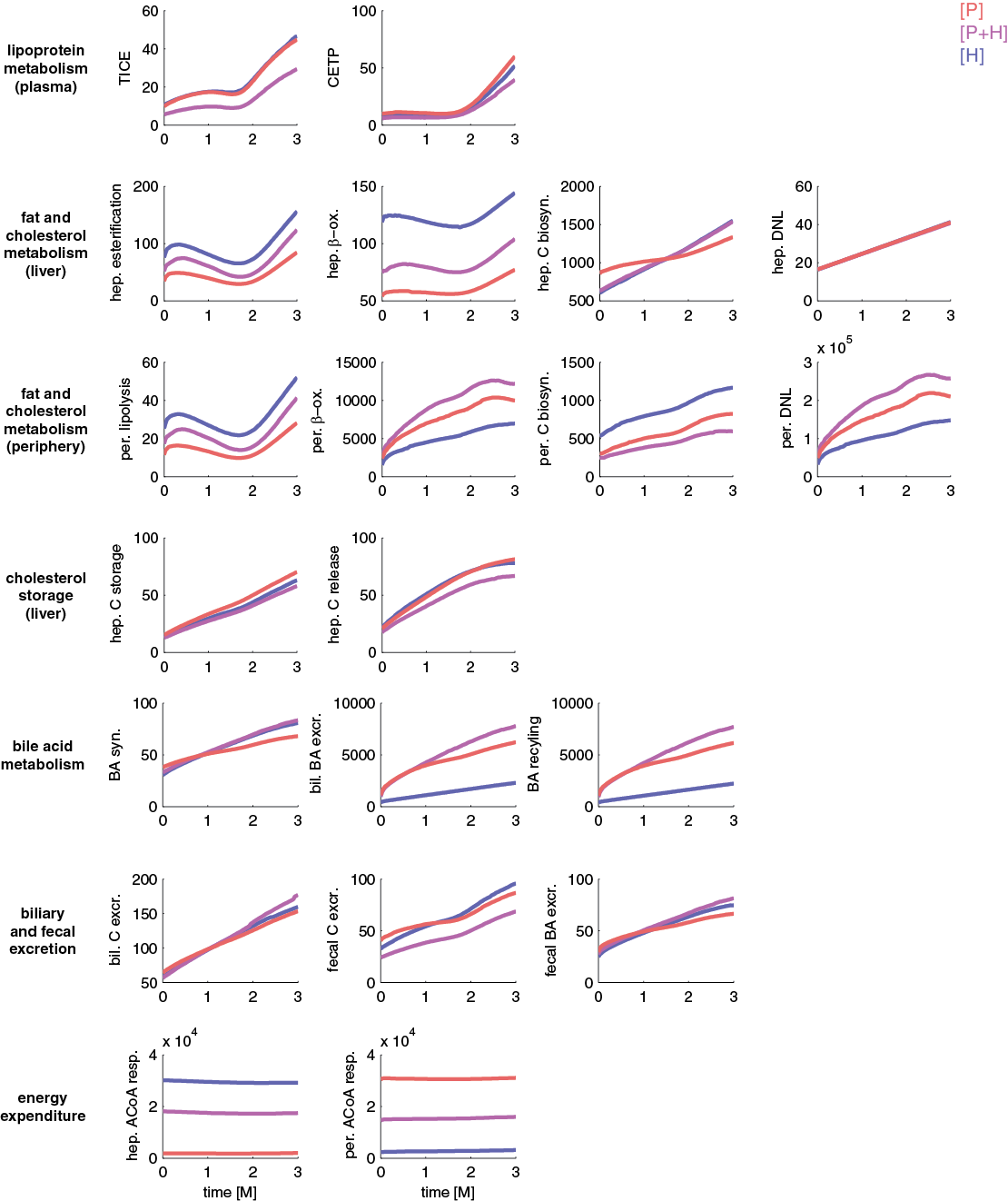


Figure S2: Metabolic fluxes depend on where the majority of energy is consumed.

The mean fluxes in individuals with predominantly peripheral energy expenditure [P] are depicted in red; mean fluxes in individuals with predominantly hepatic energy expenditure [H] in blue; and mean fluxes of individuals with both peripheral and hepatic energy expenditure [P+H] in purple. All fluxes are expressed in µmol/day.
