# Supplementary material for: Computational modelling of energy balance in individuals with Metabolic Syndrome
Source: BMC Syst Biol. 2019 Feb 26;13:24. doi: 10.1186/s12918-019-0705-z (PMC6390597; doi:10.1186/s12918-019-0705-z)
Supplement: Supplementary file 3 — Table S2. Relative contribution of substrate oxidation to peripheral and hepatic energy expenditure. The relative contribution of substrate oxidation is depicted as mean ± standard deviation, and the minimum and maximum bounds are denoted between brackets. The number of virtual individuals adhering to the physiological bound of at least 57% fat oxidation is highlighted in grey. (DOCX 16 kb) [file 12918_2019_705_MOESM3_ESM.docx]

Additional file 3: Table S2: Relative contribution of substrate oxidation to peripheral and hepatic energy expenditure.

The relative contribution of substrate oxidation is depicted as mean ± standard deviation, and the minimum and maximum bounds are denoted between brackets. The number of virtual individuals adhering to the physiological bound of at least 57% fat oxidation is highlighted in grey.

|  | peripheral energy expenditure | | | |  | hepatic energy expenditure | | | |
| --- | --- | --- | --- | --- | --- | --- | --- | --- | --- |
|  | carbohydrate oxidation [%] | fat oxidation [%] | | protein oxidation [%] |  | carbohydrate oxidation [%] | fat oxidation [%] | | protein oxidation [%] |
|  |  |  |  |  |  |  |  |  |  |
| [P] | 75.1 ± 21.8  [5.1 - 100] | 15.4 ± 24.1  [7.3e-10 - 95] | ≥57%: N=32 (9%) | 9.6 ± 5.4  [6.7e-09 - 20] |  | 32.6 ± 26.7  [1.8e-08 - 100] | 33.5 ± 29.4  [0.049 - 100] | ≥57%: N=103 (29%) | 33.9 ± 28.3  [3.1e-11 - 100] |
|  |  |  |  |  |  |  |  |  |  |
| [P+H] | 58.1 ± 29.4  [0.59 - 99] | 23.4 ± 28.8  [5.1e-6 - 99] | ≥57%: N=21 (15%) | 18.5 ± 19.6  [8e-06 - 58] |  | 80.8 ± 20.7  [33 - 100] | 1.8 ± 2.9  [0.017 - 23] | ≥57%: N=0 | 17.3 ± 19.6  [1e-7 - 58] |
|  |  |  |  |  |  |  |  |  |  |
| [H] | 29.2 ± 20.5  [0.0015 - 93] | 40.5 ± 36.1  [7.7e-11 - 100] | ≥57%: N=63 (34%) | 9.6 ± 5.8  [4e-12 - 90] |  | 89.4 ± 5.7  [74 - 99] | 1.0 ± 1.0  [0.012 - 9.7] | ≥57%: N=0 | 9.6 ± 5.8  [5.3e-12 - 21] |
|  |  |  |  |  |  |  |  |  |  |
